# Modeling Amyloid-Beta as Homogeneous Dodecamers and in Complex with Cellular Prion Protein

**DOI:** 10.1371/journal.pone.0049375

**Published:** 2012-11-08

**Authors:** Steven L. Gallion

**Affiliations:** Wallingford, Connecticut, United States of America; National Institute for Agricultural Research, France

## Abstract

Soluble amyloid beta (Aβ) peptide has been linked to the pathology of Alzheimer’s disease. A variety of soluble oligomers have been observed to be toxic, ranging from dimers to protofibrils. No tertiary structure has been identified as a single biologically relevant form, though many models are comprised of highly ordered β-sheets. Evidence exists for much less ordered toxic oligomers. The mechanism of toxicity remains highly debated and probably involves multiple pathways. Interaction of Aβ oligomers with the N-terminus of the cellular form of the prion protein (PrP^c^) has recently been proposed. The intrinsically disordered nature of this protein and the highly polymorphic nature of Aβ oligomers make structural resolution of the complex exceptionally challenging. In this study, molecular dynamics simulations are performed for dodecameric assemblies of Aβ comprised of monomers having a single, short antiparallel β-hairpin at the C-terminus. The resulting models, devoid of any intermolecular hydrogen bonds, are shown to correlate well with experimental data and are found to be quite stable within the hydrophobic core, whereas the α-helical N-termini transform to a random coil state. This indicates that highly ordered assemblies are not required for stability and less ordered oligomers are a viable component in the population of soluble oligomers. In addition, a tentative model is proposed for the association of Aβ dimers with a double deletion mutant of the intrinsically disordered N-terminus of PrP^c^. This may be useful as a conceptual working model for the binding of higher order oligomers and in the design of further experiments.

## Introduction

The amyloid beta protein (Aβ) is central to the pathology of neurodegenerative diseases such as Alzheimer’s. High levels of Aβ oligomerization in the disease state leads to plaque deposits consisting of insoluble β-sheet fibrils. The pathway for oligomerization and eventual fibril formation is complex and only partially characterized [1]. Much of the difficulty in delineating the process is due to the high degree of tertiary and quaternary conformational heterogeneity exhibited by Aβ as well as experimental challenges in isolating consistent, physiologically relevant forms.

It has been demonstrated that soluble Aβ oligomers correlate with the degree of neurotoxicity and cognitive impairment whereas the level of monomeric Aβ or insoluble fibrils do not [2]. Soluble oligomers are a heterogeneous, dynamic distribution of aggregates typically comprised of 2–14 monomers, as well as higher order structures [3]–[6]. Toxicity has been proven in a wide variety of oligomer sizes [7]–[9]. Native dimers and trimers have been shown to be among the most potent toxic species [8], [10]. The dodecameric form of Aβ42 has been indicated in a number of independent studies as a key toxic species. Examples include a 56 kDa oligomer isolated from transgenic mice [11], detergent-solubilized “globulomers” [6], derived from synthetic Aβ42, and a subpopulation of Aβ-Derived Diffusable Ligands (ADDLs) that exist as combinations of 3–24 monomeric units [12]. All dodecameric structures are approximately spherical with diameters of 40–50 Å.

One proposed mechanism for the toxicity of Aβ oligomers is through specific binding to the cellular isoform of the prion protein, PrP^c^ [13]. The prion protein is widely expressed on the surface of neurons, with a glycosylphosphatidylinositol anchor to the cell membrane at the C-terminus, a structured portion from 121–231 involving a short 2-stranded, antiparallel β-sheet (S1 and S2) and 3 α-helices (A-C). The N-terminus is natively disordered. The original studies located the Aβ oligomer binding site within the unstructured region, between residues 95–105 [13]. Subsequent studies have confirmed the importance of residues in this vicinity [14], [15]. The interaction between ADDLs and PrP is prevented by antibodies to the primary binding region as well as to helix A. [14] Binding affinities determined with surface plasmon resonance (SPR) for a series of deletion mutants further quantified the relative contributions of residues in the N-terminus to oligomer binding [15]. No loss of affinity was noted after removal of the octapeptide repeat (51–91) or the hydrophobic (111–125) segments but a significant decrease in affinity was observed with removal of the basic residue cluster ^23^KKRPK^27^. Only minor loss of affinity occurred on deletion of residues 101–110. Together, this data seem to implicate a small number residues near the N-terminus of the primary binding region contribute the most to the high affinity.

Whether via PrP^c^ or some other mechanism, Aβ oligomers bind specifically to neurons and block long term potentiation. For descriptive clarity, in this study Aβ42 residues are divided into 3 regions: N-terminus (1–17), central (18–30) and C-terminus (31–42). The C-terminus of synthetic globulomers appears to be excluded from solvent since it is resistant to proteolysis and exhibits low amide hydrogen-deuterium (H/D) exchange [6]. These globulomers do not react with antibody specific for the C-terminus, suggesting that all monomers have similar fold with buried C-termini [6]. The basic sidechain of K28 was found to be protected from crosslinking and circular dichroism measurements supported the presence of β structure [6]. A NMR study of the Aβ40 preglobulomer (a tetrameric precursor to globulomers) proposed dimeric units with central residues in an intrastrand antiparallel β-sheet and C-terminal residues in an interstrand parallel β-sheet [16].

The presence of high β-sheet content in oligomers is not ubiquitous. Stable, soluble oligomers of synthetic Aβ42 have characterized under low salt and temperature conditions [17]. The primary species were described as pentamers or hexamers with decamers or dodecamers present in lower amount. The pentamer or hexamer forms rapidly dimerized under physiological conditions and had no detectable β-sheet structure. Solvent accessible turns were noted for H13–Q15, G37–G38 and G25–G29. The turn at G25–G29 (“central turn”) facilitates close contact between residues Phe 19 and Leu 34, evidenced by a strong NOE cross-peak. Interestingly, the turn at G37–G38 (“C-terminal turn”) was not found in the preglobulomer NMR structure, but does exist (with high mobility) in a NMR structure of Aβ40 bound to an artificial antibody [18]. The presence of a C-terminal turn in oligomers was confirmed in NMR studies of analogues in which D-proline is substituted for G37 [19]. Importantly, these rigid analogues stabilized soluble, synthetic oligomers under physiological conditions and, similar to the results of Ahmed, et al, no β-sheet or α-helical structure was noted. Additionally, simulations of C-terminal peptides such as Aβ(30–42), Aβ(31–42) co-assemble into Aβ42 oligomers and have low β-sheet propensity [20], [21].

The recent crystal structure of the Aβ18-41 fragment expressed with single variable chain immunoglobulin shows a tetrameric arrangement [22]. Since tetramers have been indicated as an intermediate in dodecamer formation, [6], [16] the structure was considered a potential model system for globulomer formation. The fold differs from monomeric and fibril structures as well as soluble oligomer structures. Unlike these structures, residues V24–N27 assume a 3_10_ helix. The sidechain of K28 interacts primarily with mainchain carbonyls rather than forming a salt bridge with D23 as noted in fibrils. A β-hairpin exists for I32–I41, unlike the parallel cross-strand orientation in the fibril or the preglobulomer. A C-terminal β-turn is well defined, and the sidechain of L34 is located ∼8 Å from F19, as these residues are located on opposite ends of a 3-stranded β-sheet. In addition, dodecamer models using the tetramer unit generate cylindrical structures rather than the spherical shapes uniformly observed in dodecameric assemblies.

These studies exemplify the wide structural variation among morphologically similar oligomers, perhaps in large part to the high sensitivity of the protein to experimental conditions [23]. The physiological relevant forms are likely in dynamic equilibrium, including both “on-pathway” and “off-pathway” to fibril formation [24]. Common elements include buried C-termini and a loop near the D23–K28 segment. In this work, the stability of a dodecameric assembly is examined in which no intermolecular β-sheet structure exists. Monomers have a single β-hairpin for I32–I41 and the sidechains of F19 and L34 are assumed to be in close proximity with no intervening β-hairpin. In addition, the N-termini (often ignored in oligomer modeling) of dodecameric chains are initially set to a α-helical conformation to assess the effect on local and global stability. While the extent of helicity is subject to debate, helical N-termini are observed by solution phase NMR studies of monomers under polar conditions, [25], [26] in protein folding simulations of oligomers [27], [28] and is observed transiently in FTIR spectra of globulomers. During molecular dynamics (MD) simulations the models show preservation of experimental constraints with respect to solvent accessibility, size, organization and contacts and may represent part of the overall ensemble of toxic dodecameric structures accessible by Aβ42.

Potential modes of interaction of Aβ42 with PrP are also explored in the current study. A model of ICSM-18: PrP^c^:ADDL ternary complex has been described, though not in detail [14]. The premise was that multiple PrP binding sites exist on the ADDL particle and require PrP:PrP interactions. These PrP:PrP interactions are assumed to be disrupted with the binding of antibody to helix A. The PrP^c^ N-terminus (extended only to residue 95) was modeled as a random coil and a spherical ADDL oligomer was constructed of 57 collapsed extended β-strands. Copies of PrP^c^:ICSM-18 were manually placed to encircle the oligomer. In the current study, residues 51–91 and 111–125 are eliminated from the N-terminus, following the results of deletion studies [15], and homology modeling is used to generate plausible, non-coil, conformations. Aβ42 dimers are then docked to the PrP^c^ model. The resulting complexes are examined for consistency with previous observations and to suggest additional experiments to further elucidate specific binding interactions.

## Materials and Methods

The crystal structure of the tetramer provides a high resolution starting structure, elements of which may be stable within a dodecamer. To construct the dodecamers, a systematic assembly process is performed using rigid protein-protein docking using monomers of Aβ17-42. Each resulting set of dimers, hexamers and dodecamers is evaluated for consistency with experimental observations.

Several experimental observations were used to guide model construction. While significant structural variation exists for proposed dodecameric models, it is generally accepted that C-terminal residues form a hydrophobic core, while N-terminal residues are solvent exposed. The globulomer appears to be a dimer of hexamers, approximately spherical with a diameter of 40–60 Å. The formation of a dimer is supported by the disruptive effect of C-terminal aggregation inhibitors that reduce average particle size by half. This suggests the stacking of two hydrophobic faces, at least partially comprised of C-terminal residues [5], [17]. At physiological pH, the carboxyl terminus will be charged, making it unlikely that it will be excluded from solvent without compensating charge neutralization. Amidation of the C-terminus leads to higher order aggregation, supporting this fact. [4] Other studies have noted the difficulty of accommodating a negatively charged terminus in a manner consistent with extensive hydrophobic packing [29]. Since oxidation of M35 blocks aggregation, it is likely that this residue is also buried [30].

The sidechains of F19 and L34 are in close proximity in a solution NMR structure of Aβ40 monomer bound to a phage display protein [18]. However, this interaction occurs across a long β-hairpin, seemingly inconsistent with the lack of any significant β-sheet noted in the previously mentioned studies [17], [19]. Furthermore, K28 is fully exposed to solvent in this structure and would be highly susceptible to cross linking agents. While a dodecameric arrangement may be conceived which provides better protection for K28 (perhaps by intermolecular salt bridging), in this work the tetramer crystal structure was used as a basis to build the monomer as K28 is more fully protected.

### Monomer Construction

Interactive modeling was performed using MOE (version 2010.10) [31]. The Amber99 force field [32] was used for all calculations. Calculations performed in the absence of explicit solvent employed the Generalized Born (GB) implicit solvent model and non-bonded interactions were scaled to zero in the range 10–12 Å.

Monomers were derived from the “A” chain of the crystallographic tetramer (PDB code: 3MOQ), corresponding to residues 18–41. Glycine at position 42 was converted to alanine, hydrogens added, and mainchain torsions adjusted to place the sidechains of F19 and L34 within 6 Å. The structure was then relaxed with a single distance constraint for the F19–L34 sidechains, using harmonic potentials to constrain intervening mainchain torsions, and tethering all other non-hydrogen atoms to crystallographic positions. A consecutive series of minimizations were performed using constraint constants of 100, 10, 1 and 0 kcal/mol/Å^2^.

While the N-terminus is less ordered, it is still important to account for its presence during docking to reduce the possibility of sterically occluded solutions. This function can be fulfilled by using a few reasonable structures. Since the first 10 N-terminal residues are typically highly disordered and likely have little contact with the rest of the molecule, the chain was first extended just for residues 11–17. These residues were initially set to either fully extended or α-helical conformation, after which conformational searches along low frequency normal modes [33] were made, keeping residues 18–42 fixed. The lowest energy model was kept from each search, resulting in 5 total monomers. Each structure was then relaxed to a root-mean-square (RMS) gradient of 0.01 kcal/mol/Å.

### Multimer Construction

Several methods have been developed to predict complexes between proteins and enhancing both the scope and accuracy of these methods remains an active area of research [34]. Where sufficient ancillary data is available to guide the docking, fast rigid body approaches based on surface complementarity can perform well. There are several protein-protein docking methods available as web services [35]. This work used GrammX [36] for generation of dimers and dodecamers and SymmDock [37] for the generation of hexamers. Both web implementations allow constraints in the search. To bias towards interactions at the C-terminus, solutions were restricted to those that had at least one pairwise interaction in this segment.

Each of the 5 monomers was used to generate a homodimer with GrammX. A total of 10 models for each dimer were examined. Models were kept only if reasonable interactions at the C-terminus were made and the interface did not occlude charged sidechains. Each dimer was then refined using the same minimization strategy applied to the monomer, followed by a 50 ps molecular dynamics simulation with distance restraints of 1 kcal/mol/Å applied to F19 and L34 sidechains, as well as mainchain hydrogen bonds. The resulting structure was then minimized to convergence at RMS gradient of 0.01 kcal/mol/Å. A total of 10 dimer models were kept for the hexamer construction.

Hexamers were generated with SymmDock by using each of the model dimers and applying 3-fold symmetry, with a restriction that the potential contact residues include the C-terminus. The same process used for dimer selection was applied for elimination and refinement of both hexamers and dodecamers, resulting in a final set of 4 dodecamers. N-termini residues 1–10 were then added as α-helices. Mainchain conformation and sidechains were manually modified to eliminate steric overlaps. Histidines were assigned a neutral charge. The final assembly was then refined with restraints, as previously described.

### MD Simulations with Explicit Solvent

Each dodecamer was placed in explicit water box using the TIP3P model [38]. Counterions were added to achieve neutrality. The system was built by performing the following sequence: (a) minimization of solvent with protein held fixed (b)5 ps heating and 20 ps solvent equilibration (c) minimization of protein and solvent with protein heavy atoms tethered to initial coordinates (d) minimization with no restraints. Energy minimizations were carried out to a RMS gradient of 0.01 kcal/mol/Å. MD simulations were performed under an NPT ensemble (1atm, 300K) with periodic boundary conditions. Bonds lengths and water geometry were constrained using the LINCS method [39] to allow a timestep of 2 fs in solving the Nosé-Poincaré-Andersen equations of motion [40]. Nonbonded interactions were scaled to zero over the range of 10 to 12 Å and long range electrostatics were treated with the reaction field method. Each system was heated from 0 to 300K over 20 ps and equilibrated for 50 ps prior to the production run of between 2 and 3.5 ns, with conformational sampling every 10 ps.

### Construction of PrP Complexes with Aβ Dimers

Dimers of Aβ42 were generated from 3 sources: (1) the first 2 chains of model M1 at the optimized endpoint of the MD simulation, (2) the crystallographic tetramer (PDB code: 3MOQ) – chain pairs AC and BD [21], and (3) 2 dimers created from the affibody-Aβ40 monomer crystal structure (PDB code: 2OTK) [18]. Dimers from the tetramer crystal structure were constructed by replacing G42 with alanine and performing constrained minimization as previously described. Preliminary tests showed that results were not significantly impacted by adding Aβ N termini in either helical or extended strand conformations. Therefore, these residues were neglected during subsequent modeling. Dimers from the Aβ40 monomer structure were created by adding residues I41 and A42 to the extracted X-ray coordinates. A C-terminal β-hairpin turn was enforced and constrained optimization performed. The resulting monomer was used to generate a dimer using GrammX, subject to the constraint that at least 2 residues in the C-terminus have intermolecular contacts with PrP residues 98–100. Dimers were formed by the stacking of the hydrophobic faces, not by intermolecular H-bonding. Interestingly, this is the same type of interaction noted in the tetramer structure [22]. Two orientations were selected: one in which the β-hairpins of the respective monomers had opposite orientations across the dimer interface (referred to as G1) and the second having the β-hairpins positioned in directional alignment across the interface (G2).

The NMR structure of human recombinant PrP(23–230) (PDB code 1QLX) [41] was used to construct a model for the complex with Aβ42. Following a constrained minimization of the initial coordinates, double-deletion mutants were built, removing residues 51–90 and 111–125. N-terminal residues 23–127 were constructed with the homology module of MOE [31] using default settings. Sidechains in the globular portion of PrP within 8 Å of M129 or L130 were allowed flexibility during refinement while all other atoms were held fixed. Final model scoring was based on atomic contact energies (ACE). ACE values are proportional to the desolvation free energy associated with the transfer of atoms from water to the protein interior [42].

### Analyses

Analysis of time-averaged properties included internal geometries, oligomer size, departure from the initial structure, atomic mobility via B-factors and solvent accessible surface areas (SASA) [43]. To compare results with H/D amide exchange data the relative SASA (rSASA) value was determined for mainchain amide protons. rSASA is defined as the ratio of the SASA for the atom in the protein to that in the reference tripeptide AxA or GxA. Each tripeptide model is set to an extended conformation. The rSASA value for the sidechain primary amine of K28 was also determined in order to compare to chemical crosslinking reactivity.

To characterize the extent of hydrophobic interactions, ACE values are calculated for non-hydrogen atoms within a 6 Å cutoff and summed for each residue. Values significantly less than zero reflect a hydrophobic environment and large positive values indicate hydrophilic or solvent exposed environments [42]. An independent means of quantifying the basis of structural stability is by examining the contributions of force field terms. To reduce noise, explicit solvent was eliminated and contributions were determined with the GB implicit solvent model. Both ACE and energy decomposition measurements were performed on the minimized trajectory endpoints.

## Results

Dodecameric assemblies created two categories of structures that appeared to be artifacts of the method. The first resulted when unrestricted docking was performed with monomers. This led to stacking of the subunits with maximum burial of the hydrophobic face as described for the tetramer crystal structure [22]. The second effect was the formation of poorly-packed dodecamers, resembling hollow spheres. This occurred mostly when the C-terminal strands were oriented in an antiparallel manner, which in turn led to concave hexamers and reduction of internal contacts in the consequent dodecamer. The formation of initial hollow, spherical dodecamers was also noted in other modeling studies [44]. This was likely a consequence of the method employed in those studies. In that work a series of rigid rotations (starting with either monomers or dimers) were used to derive the initial assembly.

Since the exploration of every possible starting configuration is beyond the scope of this work, only the most likely candidates were considered, with each complex required to have reasonable interfacial interactions. This permits faster convergence during MD tests of structural stability [45], [46]. Multimer selection was therefore restricted to favor interactions between residues in the respective C-terminal segments, interacting in a parallel fashion. This led to dodecamers that appeared to be packed well. The packing densities were measured using Voronoia [47]. All models had average values of 0.8, which is identical to the tetramer Aβ (17–42) crystal structure.

**Figure 1:**
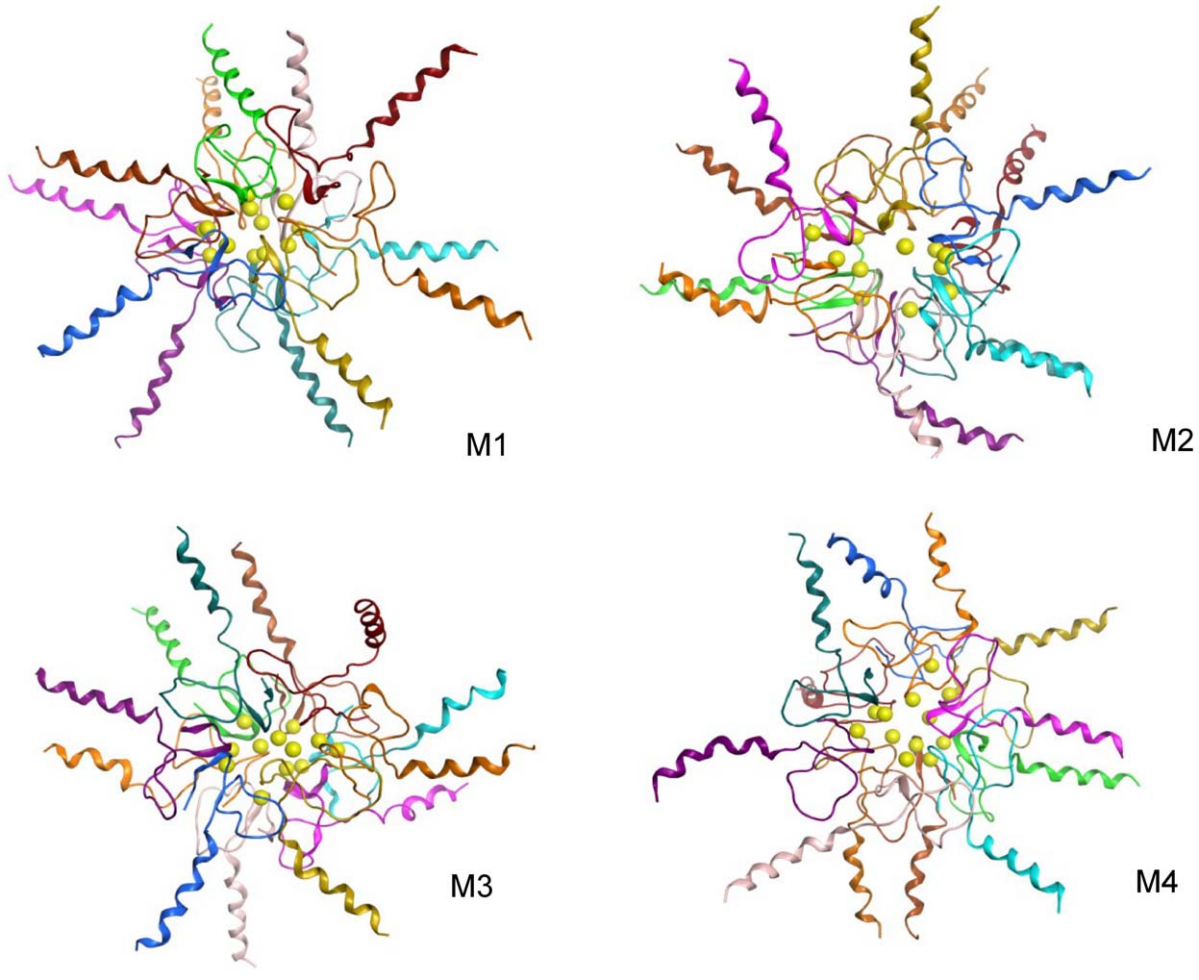
Initial structures for dodecamer models. Each dodecamer was assembled from symmetric hexamer subunits comprised of asymmetric dimers. The view is down the approximate 3-fold axis of the hexamer. C-terminal β-hairpins form a hydrophobic core and N-terminal residues are set to helical conformation. Met35 sulfur atoms are depicted as spheres. The relative position of these atoms helps to illustrate the structural variance between the models. The structures are rendered with MOE [31].

All initial models consist of a hydrophobic inner core formed by C-terminal β-strands with the C-terminal turn oriented inward (Figure 1). The negatively charged carboxyl termini are oriented towards the exterior of the assembly, permitting interactions with solvent. Turns from adjacent chains are oriented in an anti-parallel manner, though no β-sheet structure exists between chains. The sidechain of M35 is consistently buried in the center of the complex. The broad central turn between residues 27 and 30 also is quite similar for each chain. The charged sidechains in the loop are exposed to solvent, including the salt bridge between K28 and D23. The reverse side of the loop helps to sequester the hydrophobic core. Although the general topologies of monomers are similar, each complex represents a distinct packing. The root-mean-square deviation (RMSD) between any 2 dodecamers is greater than 5 Å, as measured using Cα atoms of residues 17–42. The N-terminal helical segments are quite similar, as these were added after the docking procedures. A number of variations occur within the off-diagonal patterns involving residues from the central and C-terminal turn regions. The central turn region contacts various N-terminal residues as well as residues in the C-terminus. Altogether, the models provide reasonable, alternative starting configurations for MD simulations.

### Trajectory Analysis

**Figure 2:**
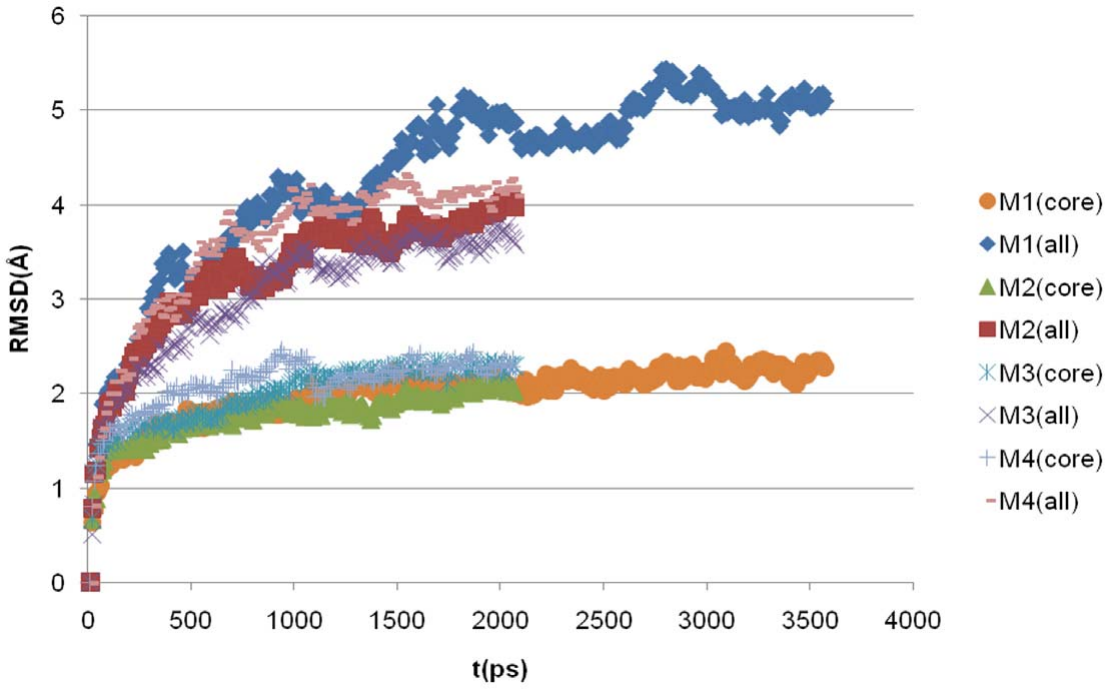
Root-mean-square deviation (RMSD) from the initial structure. Each model displays similar structural variance from the initial configuration. The RMSD for the core atoms rapidly achieves convergence of between 2 and 2.3 Å. The total RMSD shows a consistent increase in change due to the transition of the N-terminal helices to a random coil state. The trajectory of M1 was extended to 2 ns to illustrate that the equilibrium achieved by the core atoms is not impacted by N-terminal motions.

Each of the 4 dodecamer models achieved convergence rapidly. Changes in the potential energy were less than 0.2% as measured by the coefficient of variation and all time-dependent slopes were near zero. The only major structural changes were associated with the unwinding of the N-terminal helices (Figure 2). Structural changes in the core residues (17–42) were quite modest with initial folds and overall orientation of chains remaining stable. The deviations are similar to those observed in many simulations starting from well-defined crystal structures and confirm that the model building process generated reasonable conformations.

**Figure 3:**
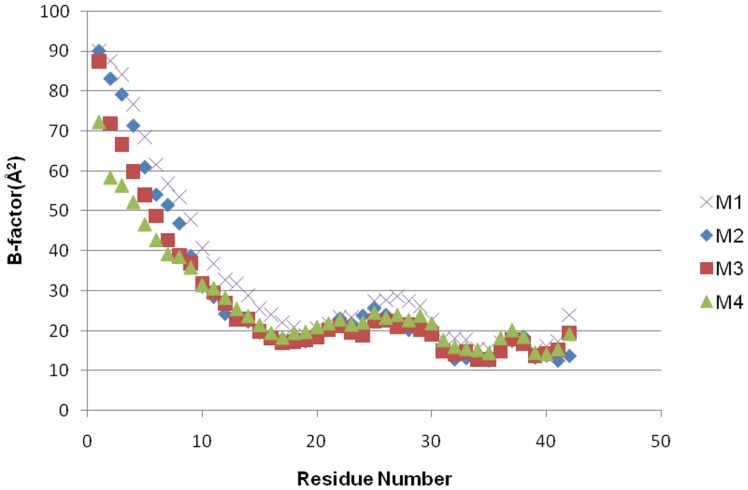
Residue mobility as determined by average B-factors. Values are averaged over all chains in the last 0.5 ns of the MD simulation. High fluctuations are noted for the unstable α helix. Residues in the core have low mobilities with central and C-terminal turn residues exhibiting higher values. The lower packing density of model M4 is reflected in significantly higher motions.

The models display similar patterns of position-dependent mainchain mobility, averaged over all 12 chains (Figure 3). The N-terminus is highly mobile, as expected, with the core residues showing much lower fluctuations. B-factor values of less than 30 Å^2^ are indicative of more ordered residues in protein crystals [48]. Model M4 has a somewhat larger overall level of mobility, suggesting a less well-packed assembly in comparison to M2 and M3. The longer sampling time of M1 yields larger fluctuations, as would be expected, although residue-based trends remain consistent for all models. Values would be expected to continue trending higher with trajectory extension, though the results are consistent with a stable core for such an assembly of non-covalent chains. Within the core, higher fluctuations occur in the turn regions, particularly that of the central-turn.

The averages of key structural features are listed in Table 1. The radius of gyration (Rg) was consistent among the models, ranging from 25–26 Å. This compares favorably with experimental values of diameters between 50 Å and 60 Å [6], [49]. The Rg for atoms contained in the core is 18–19 Å, indicating that the flexible N terminus (comprising 40% of the residues) increases total size by 30%. No significant time-dependent changes were noted as the helices transform to more random coil. The primary amino group of K28 remains sequestered from solvent, with only about 20% exposure relative to the tripeptide standard. Even while still positioned on the surface of the particle, the sidechain appears to be protected from cross-linking agents, in accordance with experiment. The distance between the sidechains of F19 and L34 remained within strong-to-moderate NOE tolerance of <7.5 Å for all models. The somewhat more mobile structure M2 resulted in the largest value of 7.2 Å, with all other models having averages <6 Å.

**Table 1:** Ensemble averages of structural features in Aβ42 dodecamer models.

Feature	M1	M2	M3	M4
Rg (1–42)(Å)	26	25	25	26
Rg (17–42)(Å)	19	18	19	18
rSASA (K28 Nζ)	0.27	0.26	0.19	0.23
Distance (F19–L34)(Å)	5.8	7.2	5.7	6.0

**Figure 4:**
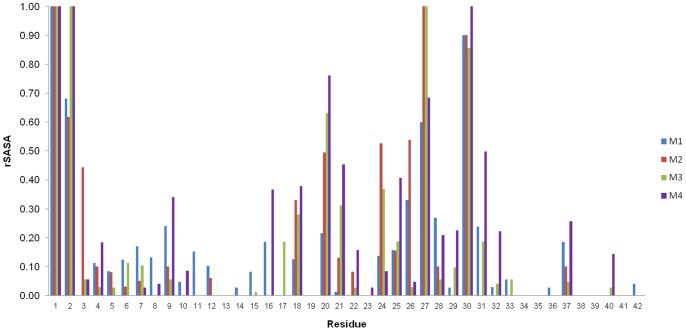
Average amide proton solvent accessible surface area relative to tripeptide (rSASA). The values are averaged over all chains. The N-terminus and central turn regions display a high degree of solvent exposure with lower values at the C-terminus, consistent with experimental H/D exchange trends. Higher values for residues in the middle of the N-terminal region would be expected as the helices transition to random coil.

Figure 4 displays the mean mainchain rSASA values, averaged over all chains. The high degree of solvent exposure is noticeable at the N terminus and turn regions. Within the hydrophobic core, residues 20, 27 and 30 have values of 50% or more relative to the tripeptide standard. The C terminal residues remain highly protected, in line with observed H/D exchange rates [17]. If amide protons with rSASA values of <10% are defined as protected, models M1–M3 show ∼60% total protection, whereas M4 remains 40% protected, compared to the 40% noted in experiments [16], [50]. Much of this is due to amide bonding within the N terminal helices. As helices transform to random coil this percentage would decline to be more in line with experimental observations.

**Figure 5:**
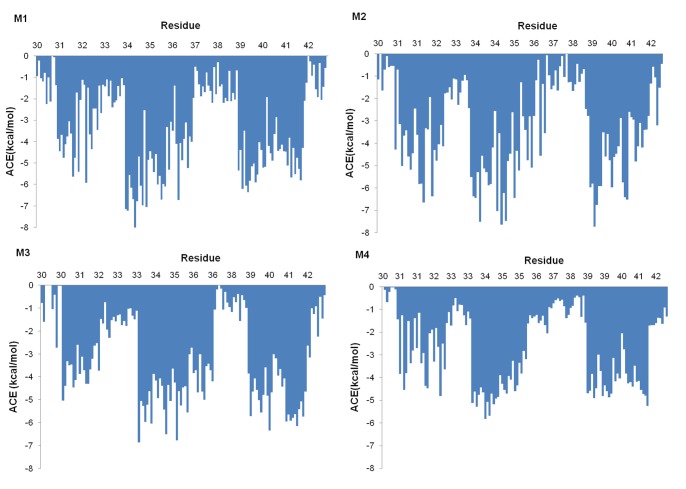
Atomic contact energies (ACE) for residues 30–42. Values are determined from minimized trajectory endpoints and shown for each chain. A negative ACE value indicates hydrophobic stabilization. Large values are noted for sidechains defining the center of the dodecamer.

The interactions within the C terminus can be characterized through independent assessment of ACE (Figure 5) and energy decomposition within the AMBER99 force field (Table 2). All ACE values are negative, with many substantial contributions supporting the existence of a hydrophobic environment in this region. Similar patterns are repeated among the models and chain-dependent variability was low with standard deviations of between 0.8 and 1.2 kcal/mol. Residues 31, 32, 34–37 and 39–41 all made significant hydrophobic interactions. The extent of hydrophobic forces in stabilizing the assembly is confirmed by comparing the hydrophobic and hydrophilic components of the force field terms shown in Table 2. The combined polar interactions (electrostatic and solvation) are nominal in comparison to van der Waals interactions between chains, regardless of model. Since atoms within the N-termini are inert in this calculation, stabilizing forces result only from the core residues.

**Table 2:** Energy contributions for core residues in optimized endpoint structures.

Model	van der Waals	Electrostatic + Solvation	Total
M1	−121	−13	−134
M2	−136	−28	−164
M3	−120	−31	−151
M4	−105	−18	−123

### PrP:Aβ42-dimer Complex

The top 10 models for the PrP N-terminus were inspected for structural trends and consistency with experimental data. As expected, a wide number of structures were formed including coil, sheet and helix. One feature appearing in a number of models was an extension of the 2 stranded antiparallel β-sheet in the PrP NMR structure. Residues 107–110 and 126 form an antiparallel strand with S1. Residues 97–101 form an additional antiparallel strand. The critical binding segment is located just prior to the reverse turn. The remainder of chain displays large variation, with a number of structures containing 1 or 2 helices and 2 or 3-stranded β-sheet. Motion of the intervening loops could easily place the important polybasic 23–27 segment in proximity to the primary binding site.

**Figure 6:**
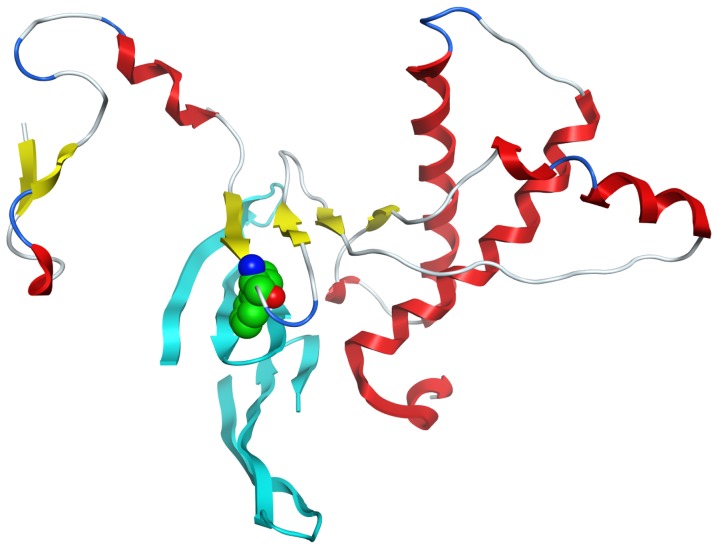
Representative model of a PrP^c^ complex with Aβ42 dimer. The N terminus of the PrP^c^ β-sheet is extended by 2 strands in a model of the double deletion mutant (Δ(51–91,111–125)). A model of Aβ42 dimer (G1) interacts with residues 97–100 of the extended β-sheet in an interfacial manner. The indole sidechain of W99 is shown for orientation. The figure is rendered with MOE [31].

Placement of Aβ42 dimers derived from the M1 dodecamer assembly or from the crystallographic tetramer did not result in compelling structures. Most of the solutions positioned the dimer such that the β-sheet was oriented orthogonal to that of the PrP^c^ sheet. Similar behavior was noted for dimer G2. However, G1 was docked in a more realistic fashion. A representative structure from the top 10 complexes is shown in Figure 6. The dimer sheet aligns approximately parallel to the PrP sheet. The sidechain of W99 is in contact with Aβ42 sidechains M35 and V40 as well near the aryl sidechain of F20. The twist in the sheet of the dimer restricts the number of sterically acceptable solutions and only small variations were noted in the top 10. The width of the 3-stranded sheet in the monomeric unit of the dimer is ∼10 Å and is accommodated well by the extended β-sheet of PrP. The reduction in the SASA on complex formation is ∼730 Å^2^, suggesting a strong interaction as the residues comprising the interface are largely hydrophobic.

## Discussion

The importance of oligomeric Aβ to memory loss in Alzheimer’s is now well established. However, the mechanism remains in question, as do the structural details and contribution of different oligomeric forms. Dodecameric assemblies have been implicated in a number of studies, but dimers and trimers also show toxicity [9], [51]. Adding to the complexity is the possibility of different aggregation pathways for fibril and non-fibrillar forms, together with the potential differences in structure and function between synthetic and endogenous oligomers of similar molecular weight [51]. This uncertainty extends to details of molecular structure. While the preponderance of evidence suggests the presence of antiparallel β-sheet in various oligomers, the extent and nature of this sheet in and between monomers varies. Another common element is a reverse turn in the central region, near residues 24–28, although both the position and structure of the turn varies [16], [50], [52]. Residues at the C-terminus portion of this turn are generally regarded as residing in an interior hydrophobic core. However, many models neglect to describe how the charged carboxylate terminus is accommodated in such an arrangement.

The current study found that a stable dodecamer could be formed involving a single β-turn configuration at the C-terminus of Aβ42. Many of the commonly observed features of globulomers are reflected in the models, including overall diameter, protection of the C-terminal amide protons from solvent exchange, exposure of the central turn region for interactions with conformationally-selective antibodies to this region, and low availability of K28 to cross-linking. The charged carboxylate terminus is oriented toward solvent and tightly packed C-terminal turns form a central hydrophobic core.

The degree to which the hydrophobic core remains stable with no extensive β-sheet structure is remarkable in comparison to the instability of the N-terminal regions that show consistent decay of initial helical structure. This supports the general experimental observation that the N-terminus lacks defined structure, though helical content is present in nascent globulomers, as measured by FTIR [50]. The similarity of the dynamic behavior in 4 independent simulations serves to validate the conclusion that hydrophobic forces between chains provides sufficient intermolecular stabilization of the dodecamer.

This contrasts with many other models that have much more extensive secondary and tertiary structural content. One highly ordered structure involves β-barrel assemblies formed by the C-terminal segment [53]. This architecture was first suggested on the basis of the similarity between the infrared spectra of synthetic oligomers and bacterial outer porins [50] and has been noted in the crystal structure of amyloid-forming protein αB crystallin [54]. The tight packing of the structure correlates well with the fact that any change to glycines in the C-terminus GxxxG motif results in lower oligomer formation and loss of toxicity. The dodecamer models described in this work also show tight packing in this region, as supported by negative ACE values, extensive surface area contacts and low rSASA values. The β-barrel models do not contain the C-terminal turn. The β-barrel model also contains a salt-bridge between the carboxylate terminus and the sidechain of K28. This would suggest that the lactam-bridged analogue would be commensurate with this β-barrel model.

Several different models of globulomers and protofibrils with substantial β-sheet structure have been recently reported [55], [56]. The most stable dodecamer model had two sets of 6-stranded parallel β-sheets. These interface at the C-terminal region in an orthogonal manner. While NMR studies of preglobulomers were consistent with a mixture of intermolecular parallel and intramolecular antiparallel structures, the presence of a C-terminal turn may decrease the probability of parallel arrangements at the C-terminus. In addition, it has been noted that the parallel orientation is not likely in prefibullar oligomers since monoclonal antibodies specific for this species do not bind to fibrillar structures with known parallel orientation [57]. FTIR data from independent studies indicate the presence of antiparallel structure in many of the soluble oligomers [50], [58]. However, the proposed parallel arrangement could represent an intermediate along the fibril formation pathway.

It is worth noting some of the differences between the dodecamers presented in this study and prior simulations of a pentamer model [56]. In those simulations, the proposed packing by Ahmed, et al led to rapid dissolution of the complex. As a consequence, monomers were constructed with a β-hairpin for residues V18–V36 and pentamers formed by extensive intermolecular parallel or antiparallel β-sheets. The resulting models were found to be stable in a 60 ns MD simulation and had reasonable correlation with H/D exchange data. Similar intermolecular stabilization is observed for the β-hairpin adopted by Aβ40 when bound to an antibody protein mimic [18]. The extensive exposure of hydrophobic surface to solvent in such pentameric β-sheets seems improbable unless shielded by the N-terminal residues.

In contrast, the current work assumes only a dodecamer species (consistent with physiological conditions), rather than independent pentamer or hexamer subunits. Independent observations of oligomers under physiological conditions also found no evidence of β-sheet structure by either CD or NMR analysis, although the C-terminal turn was present [19]. The simulations presented here test the feasibility of dodecamers in which the hydrophobic core is comprised of identical monomers that contain only a short β-hairpin formed from residues I31–I41. The results illustrate that all models have quite reasonable comparisons to experimental observations and may represent a component of the polymorphic distribution of soluble oligomers. The ability to retain initial structure without any intermolecular β-sheets demonstrates the significant contributions that hydrophobic interactions have in forming a stable oligomer. Cysteine scanning mutagenesis has found oligomer disruption resulting from changes to I31, I32, L34, V39-I41, suggesting that these sidechains (but not mainchain interactions) contribute to inter-strand contacts [59]. The structures reported here are fully consistent with this finding. In addition, the oxidation of M35 is known to prevent oligomerization [30]. All models position this sidechain within the hydrophobic core.

Another recent modeling study of Aβ globulomers started with annular arrangements of either monomeric or in-register, parallel dimeric Aβ17–42 [44]. Both monomers and dimers were created from the fibril structure [60]. This annular organization generates a hollow core, as previously described. The void was eventually filled with hydrophobic C-terminal chains during a 40 ns MD simulation, leading to occasional chain entanglement. The most stable models were noted to be formed from the dimeric subunits, demonstrating the necessity for extensive parallel hydrogen bonding between monomers in this protocol. The models presented here are also assembled from dimers, but with no hydrogen bonding between monomers. Another key difference is that no entangled peptides were detected in any model presented here. This is likely a consequence of having well-packed cores in the initial structures.

Many MD simulations of oligomers are performed over much longer timeframes than used in the current study. Many of these involve starting configurations that are far from equilibrated structures, such as in protein folding simulations. [61], [62] When disorder is low, equilibrium and convergent dynamics are achieved fairly rapidly for all but motions along low frequency normal modes or infrequent events with relatively high energy barriers [45]. The fluctuations and structural changes monitored during the simulations strongly support the achievement of local equilibrium behavior for atoms within the hydrophobic core. Indeed, the large amplitude motions associated with the dissolution of N-terminal helices are decoupled from the dynamics of the core atoms. The high mobility of the N-terminus is insufficient to disrupt the stability of the hydrophobic core. Furthermore, a continuation of the M1 trajectory led to no significant changes. Given the similarity of the ensemble averages computed from 4 different starting configurations, it seems unlikely that an infrequent event would cause sudden oligomer disruption with more extensive sampling.

The wide disparity of structural models may be reflective of the inherent polymorphic nature of soluble oligomers. While similarities exist among different characterizations, experimental data is imprecise and the population of conformers is sensitive to many factors. It is instructive to note that monoclonal antibodies raised against Aβ20–42 globulomers do not detect full length Aβ42 globulomers prepared in identical manner, but do detect Aβ42 oligomers in brain extracts from Alzheimer patients [52]. NMR data from the same group was performed on Aβ42 globulomer tetrameric subunits [16]. Detailed extrapolation of such structural data to functional results may not be trivial. Toxicity exists for oligomers exhibiting similar size and morphology but differences in secondary structure as determined by CD, FTIR and NMR. With such documented heterogeneity, it is not entirely clear that toxicity is attributable to structures determined in the unbound state or that there is a single causal effect [51], [63]–[65]. The resulting divergence of conclusions may simply reflect the underdetermined nature of the problem. If direct binding of large oligomeric complexes such as dodecamers is definitively proven it may indicate that they serve as a conformational stabilizing reservoir from which lower order oligomers diffuse for binding. The use of rigid Aβ analogues (both interstrand and intrastrand) as well as consistent experimental characterization may help to resolve this aspect.

The model of the PrP^c^:Aβ42 dimer complex also must be interpreted in the context of large uncertainties and paucity of available structural data. The proposed extension of the native β-sheet may be a reasonable possibility. Neurotoxicity is mediated by PrP^c^ for a variety of β-sheet structures and is blocked by the antibody A11 known to recognize sequence-independent β-sheet edges [57], [66], [67] PrP^c^ also forms a dimer via the β-sheet in crystal structures [68]. A similar extension of the β-sheet was noted in MD simulations possibly involving motions of helix A [69], [70]. It is also noteworthy that residues 98–110 have been found essential for PrP^Sc^ aggregation [71]. NMR studies of PrP23–144 indicate β-sheet fibrils forming from residues 112–141, but it is not clear if this result translates to full-length protein [72].

The docking simulations between PrP^c^ and Aβ dimers are limited in the scope of structures tested and absence of conformational variability. However, the hydrophobic interface with the G1 dimer model is certainly plausible. The model suggests that only residues from one monomer are involved in the interaction with PrP^c^. If this is correct, then alternating sidechains would primarily contribute directly to the interface. The removal of the W99 sidechain from solvent would require significant stabilization from Aβ residues and is worth further investigation, including monitoring fluorescence quenching on binding. A crystal structure of bovine PrP(90–105) bound to single chain Fv fragment describes the tryptophan sidechain buried deep in a hydrophobic pocket. The buried indole contributes about 30% of the total reduction in bound peptide surface area and the indole nitrogen is positioned to form H-bonds with the receptor [73]. The PrP peptide adopts a broad turn with tryptophan near the center in order to position the indole within the pocket. This conformation cannot be ruled out in PrP^c^:Aβ oligomer binding without further testing and constrained analogues may be particularly useful in this regard.

The deletion of residues 101–125 did not affect oligomer binding, so it appears that the structural events of aggregation are independent of those of oligomer binding. Residues 102–105 are a PxxP motif that would disrupt a longer β-strand. This region may form a polyproline-II helix and constitute a SH3-binding site [74]. Previous pH-dependent MD simulations led to additions of two antiparallel strands within the hydrophobic segment (115–117 and 120–122) [69]. If this occurs in the native PrP^c^ interaction with Aβ, the additional 2 strands reported here may represent a 4-strand extension that is only stabilized through binding to β-sheet ligands to reduce the solvent exposure of the hydrophobic surface. However, it is intriguing that only a fraction of the available surface area in such a complex forms specific, high-affinity, interactions with the oligomer.

The correlation of size with affinity remains in question, both for PrP^c^ and Aβ. Average oligomer size has ranged from 20 to between 50 and 100 monomers [13], [15]. The relevance of SDS-stable dimers to the active oligomeric form(s) remains in debate but antibodies raised against synthetic Aβ40(S26C) dimers aggregates have proved effective in disease models [75]. The same synthetic dimers as well as soluble dimers isolated from Alzheimer’s brain tissue have been found to be neurotoxic in cell cultures [9]. It is important to note that Aβ40(S26C) dimers have no defined structure or toxicity until aggregation occurs. The steric restrictions resulting from the disulfide link make the face-to-face stacking noted within the G1 and G2 dimers highly unlikely.

Based on the results reported here, it would useful to test a series of conformationally restricted inter- and intra-molecular dimers that would permit interfacial stacking and limit additional aggregration. In addition, alanine scans and conformationally restricted peptides of PrP(90–110) could provide further binding details as well as serve as potential peptidomimetic antagonists. Particularly useful would be N-methyl analogues to test for β-sheet disruption. One key variant is suggested by the N100G substitution in rabbit and mink PrP that abolishes 6D11 antibody binding [76]. Testing the N100A mutant would help eliminate the possibility of a conformational effect and help define the structure-affinity profile. Since W99 and N100 occupy opposite sides of the sheet in the current model, hydrogen bond contributions to affinity from the N100 sidechain may signal interfacial stacking of Aβ oligomer on both sides of the PrP^c^ β-sheet.

It is clear from the proposed β-sheet extension model that deletion of the octapeptide region would not be expected to impact oligomer binding. However, while the SPR analysis of deletion mutants found no affect attributable to this region, a separate SPR study examining the binding of oligomer to 13-mer PrP peptide arrays identified this region as having the highest affinity. Although the octapeptide region is known to bind copper ions, the binding of PrP^c^ to oligomeric Aβ is independent of copper concentration [13]. NMR studies have noted broad turns within the sequence [77]. It is conceivable that the constituent Trp, His and Gln residues assume a conformation similar to the bound conformation of Trp, His(or Lys), Asn (or Gln) residues in PrP^c^ (97–101).

The current model positions the bound Aβ dimer towards the membrane. This is a partial consequence of the docking constraint enforcing some interaction with W99, which has the sidechain oriented towards the membrane. A single change in the alignment of the β-hairpin would alter this, allowing for the approach of a much larger oligomeric structure. It is difficult to assess the probability of either orientation with the available data, though the prevention of oligomer binding with pretreatment of PrP^c^ with ICSM-18 may provide a clue [14]. In the models derived here, the ICSM-18:PrP^c^ complex is sterically compatible with dimeric Aβ even with the W99 indole oriented away from the membrane. It may however, preclude the interaction of larger oligomers. In addition, coupled motions of helix A and the loop to the first β-strand have been noted [78]. The enhanced mobility of the loop may be due to limited stabilizing interactions with the folded core. Changes in this region, such as the M205R mutant, result in higher mobility of helix A in MD studies [79]. If the hydrophobic segment interacts with this loop instead of forming additional antiparallel sheet with S1, it is possible that antibody bound to helix A results in allosteric prevention of oligomer binding to PrP^c^. Either of these possibilities does not require multiple PrP^c^ binding to the oligomer as previously proposed [14]. In addition, significant motions of this region have been noted in other MD studies of human PrP^c^(residues 125–230) [80] and in a kinetic characterization of ovarian PrP oligomerization [81]. Simulations also suggest a wide landscape of accessible Aβ dimers [82], [83]. It would be interesting to study complexes identified from simulations of fully flexible Aβ dimers and PrP^c^.

### Conclusions

Dodecameric assemblies of Aβ42 were modeled with a single secondary structure feature involving a short β-hairpin turn at the C-terminus. In classic MD simulations with explicit solvent the dodecamers retained close hydrophobic packing at the C-terminus and the β-hairpin turn remained stable, regardless of modest differences in initial structure. This was in contrast to the N-termini that were initially set to α-helical form. The dissipation of α-helical structure did not impact the structure of the hydrophobic core. The models are consistent with a range of experimental data, including size, H/D exchange rates, mutational scans, peptidomimetics, chemical reactivity and antiparallel character. The study highlights the importance of hydrophobic interactions, as compared to highly organized interstrand hydrogen bonding in conferring stability to a dodecameric assembly. In contrast to simulations of loose pentameric assemblies [56] dodecameric structures without large, rigid interstrand hydrogen bond networks are observed to be stable in simulations of similar time scale. They may provide a basis for more extensive simulations needed to demonstrate stability in more highly ordered assemblies [53], [55]. These structures may represent one of many relevant forms in a highly heterogeneous population.

The abbreviated N-terminus of the PrP^c^ structure was derived through homology modeling and used in a series of rigid protein docking studies with various dimeric forms of Aβ42. Extension of the native β-sheet was found to provide a specific interaction surface for Aβ42 dimers. The dimer exhibiting the best interaction surface was derived from monomers consisting of 2 antiparallel turns. The dimerization occurred by hydrophobic stacking rather than intermolecular H-bonding as seen in fibrils. The interaction between PrP^c^ and Aβ42 dimers does not require formation of mainchain hydrogen bonding such as noted in crystal structures of dimeric PrP^c^. The model appears consistent with current, albeit limited, experimental structural data. It may serve as a basis for further experimental examinations of the interaction of these intrinsically disordered peptides and hopefully lead to the design of compounds with therapeutic value.
